# Supplementary material for: i-Motif, not G-quadruplex, stability regulates insulin expression
Source: Nucleic Acids Res. 2026 Jan 29;54(3):gkag041. doi: 10.1093/nar/gkag041 (PMC12852952; doi:10.1093/nar/gkag041)
Supplement: gkag041_Supplemental_File [file gkag041_supplemental_file.docx]

**SUPPLEMENTARY Information**

i-Motif Stability, not G-quadruplex Stability, Modulates Insulin Expression in Reporter Gene Assays

Dilek Guneri,^1,2^ Christopher J. Morris,^1^ Yiliang Ding,^3^* Timothy D. Craggs,^4^* Steven S. Smith,^5^* Zoë A. E. Waller^1^*

1. School of Pharmacy, University College London, 29-39 Brunswick Square, London, WC1N 1AX, UK.
2. School of Health, Science and Society, University of Suffolk, 19 Neptune Quay, Ipswich, IP4 1QJ, UK.
3. Department of Cell and Developmental Biology, John Innes Centre, Norwich Research Park, Norwich NR4 7UH, UK
4. The University of Sheffield, Department of Chemistry, Western Bank, Sheffield, UK.
5. Department of Stem Cell Biology and Regenerative Medicine, Beckman Research Institute of the City of Hope, 1500 E. Duarte Road, Duarte CA 91010-3000, USA

*Corresponding Authors: [z.waller@ucl.ac.uk](mailto:z.waller@ucl.ac.uk); [SSmith@coh.org](mailto:SSmith@coh.org) [Yiliang.Ding@jic.ac.uk](mailto:Yiliang.Ding@jic.ac.uk); [t.craggs@sheffield.ac.uk](mailto:t.craggs@sheffield.ac.uk)

**Table S1.** Naturally occurring ILPR variant (indicated e.g. 1C) and mutant sequences (indicated as e.g. 1C_mut_). Loop regions are underlined, changes from the original sequence are indicated in bold, nucleotide deletions are indicated by a dash (-). Most prominent UV melting temperature, structural characteristics from thermal difference spectra (TDS) at pH 5.5, and transitional pH (pH_T_), and. UV were repeated in triplicate and data are shown as mean±SD (n = 3) where SD is below 1.0°C. iM = i-motif; Mix = Mixed species of i-motif and hairpin; Hp = hairpin; nt = no transition observed; nss = no secondary structure observed. Sequences with two transitional pH values are indicated as pH_T1_/pH_T2_.

| **Label** | **Sequence** | **T_M_ (°C)**  **at 295 nm** | **TDS Profile at pH 5.5** | **pH_T_** |
| --- | --- | --- | --- | --- |
| 1C | TGTCCCCACACCCCTGTCCCCACACCCCTGT | 55 | iM | 6.6 |
| 1C_mut_ | TGTCCCC**T**C**T**CCCCTGTCCCC**T**C**T**CCCCTGT | 60 | iM | 6.9 |
| 1C_mut2_ | T**T**TCCCCACACCCCT**T**TCCCCACACCCCT**T**T | 55 | iM | 6.7 |
| 1C_mut3_ | TGTCCCC**TTT**CCCCTGTCCCC**TTT**CCCCTGT | 60 | iM | 6.8 |
| 1C_mut4_ | TGT**T**CCCACA**T**CCCTGT**T**CCCACA**T**CCCTGT | 49 | iM | 6.3 |
| 1C_mut5_ | TGTC**T**CCACAC**T**CCTGTC**T**CCACAC**T**CCTGT | 32 | iM | 6.0 |
| 1C_mut6_ | TGTCC**T**CACACC**T**CTGTCC**T**CACACC**T**CTGT | 29 | iM | 5.6 |
| 1C_mut7_ | TGTCCC**T**ACACCC**T**TGTCCC**T**ACACCC**T**TGT | 44 | iM | 6.1 |
| 1C_mut8_ | TGT**T**C**T**CACA**T**C**T**CTGT**T**C**T**CACA**T**C**T**CTGT | nt | nss | nt |
| 1C_mut9_ | TGTC**T**C**T**ACAC**T**C**T**TGTC**T**C**T**ACAC**T**C**T**TGT | nt | nss | nt |
| 1C_mut10_ | TGT**T**C**T**CACACCCCTGT**T**C**T**CACACCCCTGT | 31 | iM | 5.9 |
| 1C_mut11_ | TGTCCCCACAC**T**C**T**TGTCCCCACAC**T**C**T**TGT | 30 | iM | 5.7 |
| 1C_mut12_ | TGTCCC**-**ACACCCCTGTCCC**-**ACACCCCTGT | 49 | iM | 6.9/6.0 |
| 1C_mut13_ | TGTCCCCACACCC**-**TGTCCCCACACCC**-**TGT | 46 | iM | 6.3 |
| 1C_mut14_ | TGTCCC**-**ACACCC**-**TGTCCC**-**ACACCC**-**TGT | 40 | iM | 6.0 |
| 1C_mut15_ | TGTCC**--**ACACC**--**TGTCC**--**ACACC**--**TGT | nt | z-DNA | nt |
| 1C_mut16_ | TGTCC**--**ACACCCCTGTCC**--**ACACCCCTGT | 36 | iM | 5.7 |
| 1C_mut17_ | TGTCCCCACACC**--**TGT**--**CCACACCCCTGT | nt | iM | 5.5 |
| 1C_mut18_ | T**-**TCCCCACACCCCT**-**TCCCCACACCCCT**-**T | 54 | iM | 6.7 |
| 1C_mut19_ | **-**GTCCCCACACCCC**-**GTCCCCACACCCC**-**GT | 58 | iM | 6.9 |
| 1C_mut20_ | TG**-**CCCCACACCCCTG**-**CCCCACACCCCTG**-** | 53 | iM | 6.5 |
| 1C_mut21_ | **-**G**-**CCCCACACCCC**-**G**-**CCCCACACCCC**-**G**-** | 54 | iM | 6.7 |
| 1C_mut22_ | TGTCCCC**-**CACCCCTGTCCCC**-**CACCCCTGT | 58 | iM | 6.7 |
| 1C_mut23_ | TGTCCCCAC**-**CCCCTGTCCCCAC**-**CCCCTGT | 59 | iM | 6.7 |
| 1C_mut24_ | TGTCCCC**-**C**-**CCCCTGTCCCC**-**C**-**CCCCTGT | 58 | iM | 6.7 |
| 1C_mut25_ | TGTCCCCA**-**ACCCCTGTCCCCA**-**ACCCCTGT | 53 | iM | 6.6 |
| 2C | T**A**TCCCCACACCCC**TAT**CCCCACACCCCT**A**T | 57 | iM | 6.7 |
| 2C_mut_ | T**A**TCC**A**CACACCCC**TAT**CC**A**CACACCCCT**A**T | 40 | Mix | 6.7/5.8 |
| 2C_mut2_ | T**A**TCCCCACACCCC**TAT**CC**A**CACACCCCT**A**T | 43 | iM | 6.2/5.8 |
| 2C_mut3_ | T**A**TCC**A**CACACCCC**TAT**CCCCACACCCCT**A**T | 40 | iM | 6.0 |
| 2C_mut4_ | T**A**TCC**G**CACACCCC**TAT**CC**G**CACACCCCT**A**T | 40 | iM | 6.2/5.6 |
| 2C_mut5_ | T**A**TCC**T**CACACCCC**TAT**CC**T**CACACCCCT**A**T | 42 | iM | 6.0 |
| 3C | TGTCCCCA**G**ACCCCTGTCCCCA**G**ACCCCTGT | 50 | iM | 6.2 |
| 3C_mut_ | TGTCCCCA**GG**CCCCTGTCCCCA**GG**CCCCTGT | 52 | iM | 5.8 |
| 4C | TGTCC**T**CACACCCCTGTCC**T**CACACCCCTGT | 40 | iM | 6.1 |
| 4C_mut_ | TGT**T**CCCACACCCCTGT**T**CCCACACCCCTGT | 50 | iM | 6.4 |
| 4C_mut2_ | TGTC**T**CCACACCCCTGTC**T**CCACACCCCTGT | 40 | iM | 6.5/5.7 |
| 4C_mut3_ | TGTCCC**T**ACACCCCTGTCCC**T**ACACCCCTGT | 48 | iM | 6.5/5.7 |
| 4C_mut4_ | TGTCCCCACA**T**CCCTGTCCCCACA**T**CCCTGT | 53 | iM | 6.7/5.9 |
| 4C_mu5_ | TGTCCCCACAC**T**CCTGTCCCCACAC**T**CCTGT | 44 | iM | 6.7/5.9 |
| 4C_mut6_ | TGTCCCCACACC**T**CTGTCCCCACACC**T**CTGT | 37 | iM | 5.9 |
| 4C_mut7_ | TGTCCCCACACCC**T**TGTCCCCACACCC**T**TGT | 44 | iM | 6.0 |
| 5C | TGTCC**T**CA**G**ACCCCTGTCC**T**CA**G**ACCCCTGT | 32 | iM | 5.4 |
| 6C | TGT**G**CCCACACCCCTGT**G**CCCACACCCCTGT | 50 | Hp | 5.2 |
| 6C_mut_ | TGT**C**CCCACACCCCTGT**G**CCCACACCCCTGT | 49 | Mix | 6.0 |
| 6C_mut2_ | TGT**G**CCCACACCCCTGT**C**CCCACACCCCTGT | 49 | iM | 6.0 |
| 6C_mut3_ | TGT**A**CCCACACCCCTGT**A**CCCACACCCCTGT | 46 | iM | 6.0 |
| 7C | TGTCCCCA**GGA**CCCTGTCCCCA**GGA**CCCTGT | nt | Hp | 5.1 |
| 7C_mut2_ | TGTCCCCA**GGA**CCCTGTCCCCA**GG**CCCCTGT | 41 | Mix | 5.8/5.0 |
| 7C_mut3_ | TGTCCCCA**GG**CCCCTGTCCCCA**GGA**CCCTGT | 48 | Mix | 5.5 |
| 8C | TGTCCTCA**G**ACCCCTGTCCTCA**G**ACCCCTGT | 32 | iM | 5.4 |
| 9C | TGTCCCC**GG**ACCCCTGTCCCC**GG**ACCCCTGT | 60 | iM | 5.2 |
| 10C | TGTCCCC**GGG**ACCCCTGTCCCC**GGG**ACCCCTGT | nt | Hp | 4.6 |

**Table S3** Biophysical characterisation UV melt and anneal and thermal difference spectrum of ILPR C1 and C6 and the complementary ILPR G1 and G6 in presence of 2.8 mM or 16.2 mM glucose or G6P in 10 mM sodium cacodylate 100 mM potassium chloride at pH 5.5 for the C-rich sequences and pH 7.0 for the G-rich sequences. (- = not detected, T_M_ = Melting Temperature, T_A_= Annealing Temperature, i-Motif = iM, Hairpin = Hp, Mix = i-motif/Hairpin mix or G-quadruplex/Hairpin mix).

| ILPR | Buffer additives | UV spectroscopy  Melting, Annealing Temperatures (°C) | | | | TDS |
| --- | --- | --- | --- | --- | --- | --- |
|  |  | 295 nm | | 260 nm | | Structure |
|  |  | *T*_M_ | *T*_A_ | *T*_M_ | *T*_A_ |  |
| 1C | None | 55±0.2 | 53±0.6 | 56±0.0 | 53±0.6 | iM |
|  | 2.8 mM Glucose | 55±0.0 | 51±1.5 | 55±0.6 | 50±0.6 | iM |
|  | 16.2 mM Glucose | 54±1.0 | 50±0.0 | 55±0.0 | 50±0.0 | iM |
|  | 2.8 mM G6P | 42±0.0 | 38±0.0 | 42±0.6 | 38±0.5 | Mix |
|  | 16.2 mM G6P | 34±1.0 | 29±0.6 | 34±0.0 | 29±0.7 | Mix |
| 6C | None | 50±0.6 | 44±0.6 | 50±0.7 | 44±0.0 | Hp |
|  | 2.8 mM Glucose | 48±0.3 | 46±0.6 | 41±0.6 | 35±0.0 | Mix |
|  | 16.2 mM Glucose | 68±0.6 | 63±0.0 | 68±0.6 | 62±1.0 | iM |
|  | 2.8 mM G6P | 39±1.0 | 35±0.6 | 40±1.0 | 35±0.0 | Mix |
|  | 16.2 mM G6P | - | - | 42±0.0 | 40±0.7 | Hp |
| 1G | None | 76±0.6 | 60±0.0 | 77±0.6 | 60±0.6 | G4 |
|  | 2.8 mM Glucose | 75±0.6 | 60±0.0 | 74±0.0 | 60±0.0 | G4 |
|  |  | 84±1.0 | 80±0.6 | 85±1.0 | 80±1.0 |  |
|  | 16.2 mM Glucose | 59±1.0 | 53±0.0 | 60±0.6 | 54±1.0 | G4 |
|  |  | 79±1.0 | 73±0.6 | 80±0.4 | 74±0.6 |  |
|  | 2.8 mM G6P | 87±0.6 | 80±1.0 | 87±1.5 | 81±1.2 | G4 |
|  | 16.2 mM G6P | 86±1.5 | 75±0.7 | 86±0.6 | 75±0.6 | G4 |
| 6G | None | 56±0.0 | 50±0.0 | 56±0.6 | 51±0.6 | Mix |
|  | 2.8 mM Glucose | 33±1.0 | 20±0.0 | 82±0.7 | 77±0.7 | Mix |
|  |  | 47±0.6 |  |  |  |  |
|  | 16.2 mM Glucose | 72±1.0 | 66±.0 | 71±1.0 | 64±1.0 | G4 |
|  | 2.8 mM G6P | 70±0.5 | 60±1.0 | 71±1.0 | 60±0.6 | G4 |
|  | 16.2 mM G6P | 70±0.0 | 60±1.2 | 69±0.6 | 59±1.0 | G4 |


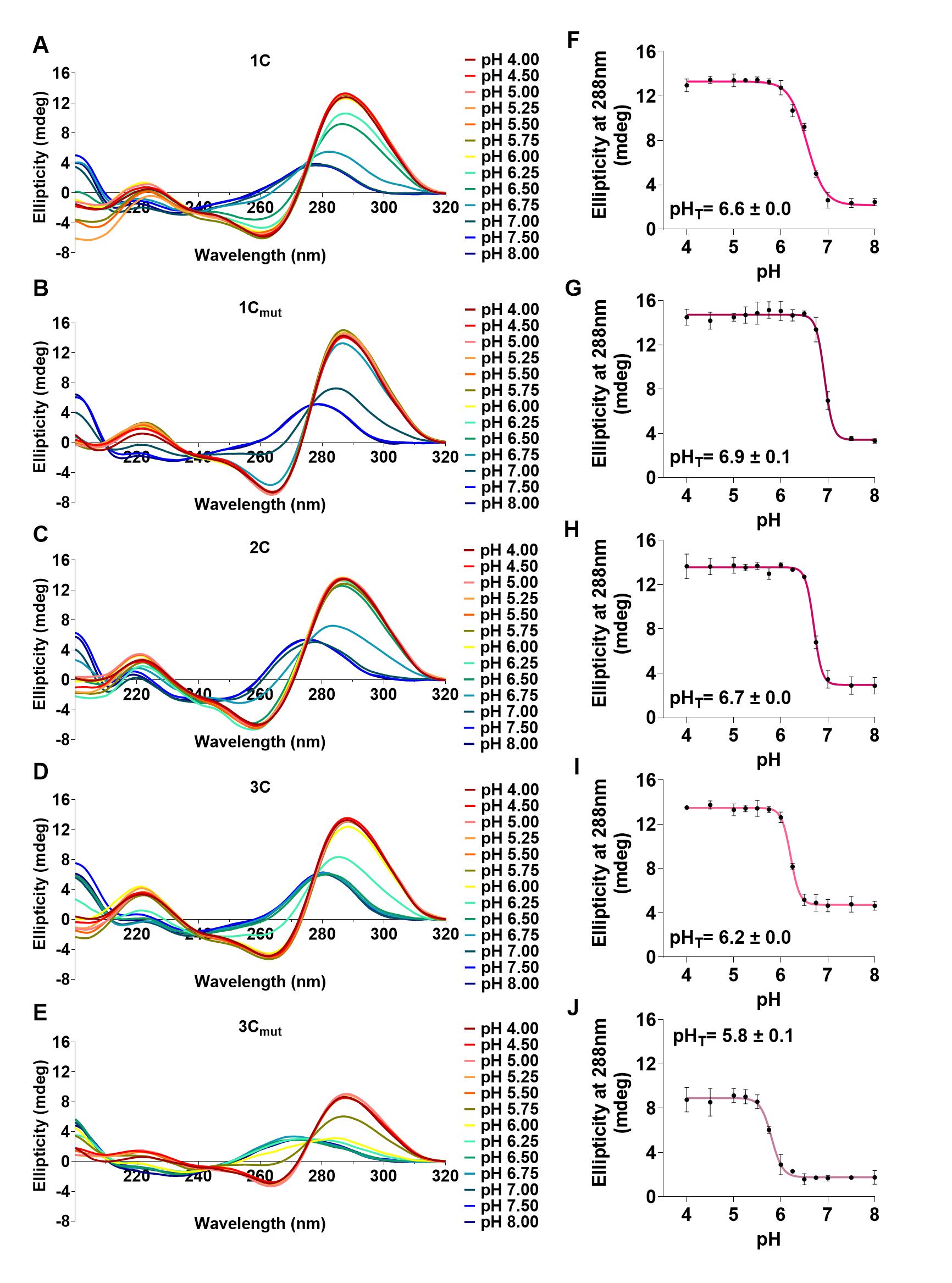


**Figure S1.** A-E) CD spectroscopy of C-rich ILPR sequences 1C, 1C_mut_, 2C, 3C, 3C_mut_. 10 µM DNA in 10 mM NaCaco 100 mM KCl and pH as indicated. F-J) Corresponding plot ellipticity of the experimental repeats (n=3, mean ± SD) at 288 nm at the measured pH conditions to determine transitional pH (pH_T_) from the inflection point of the Boltzmann sigmoidal curve. Source data for this figure are provided as a Source Data file.


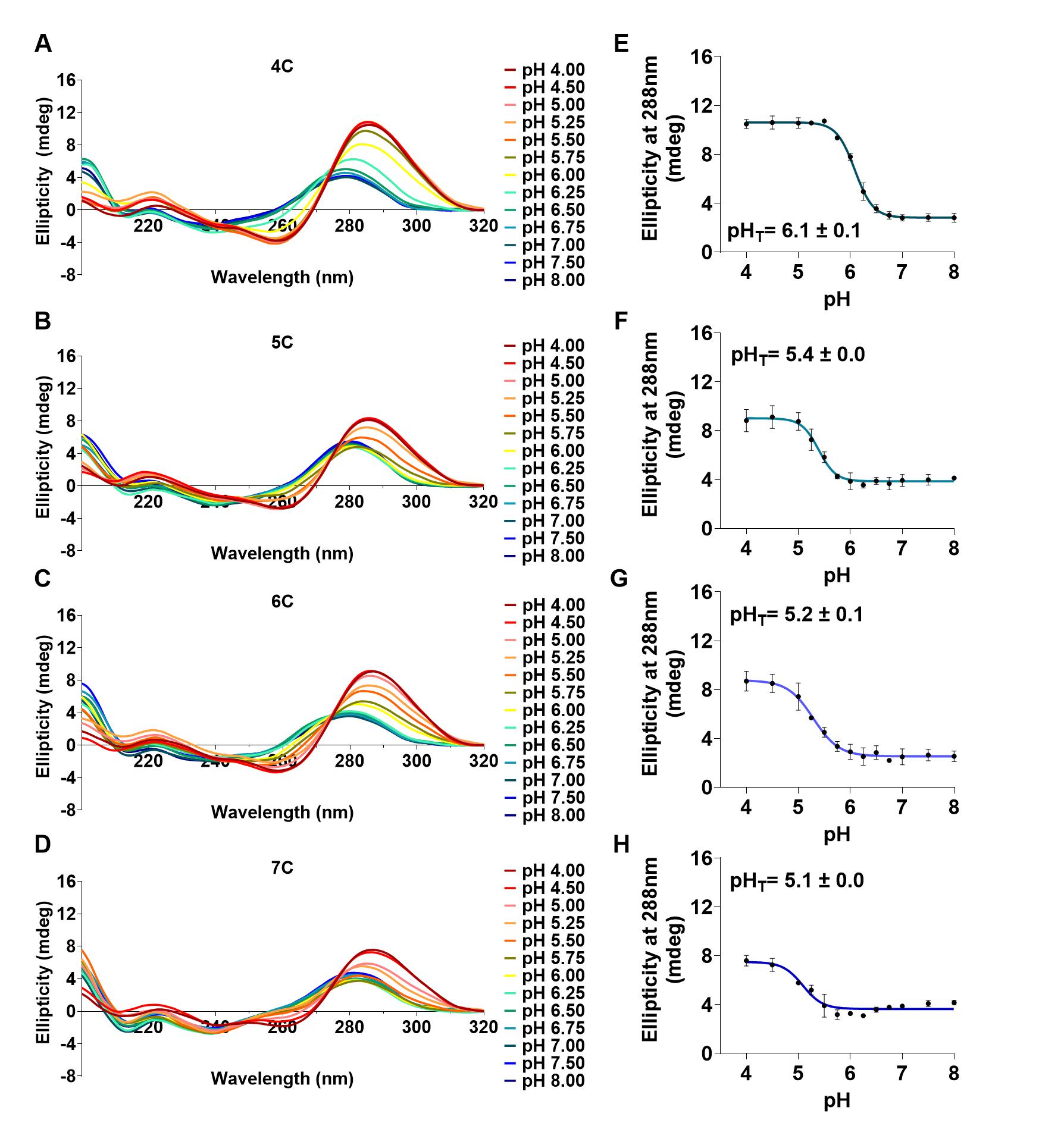


**Figure S2.** A-D) CD spectroscopy of C-rich ILPR sequences 4C, 5C, 6C, and 7C. 10 µM DNA in 10 mM NaCaco 100 mM KCl and pH as indicated. E-H) Corresponding plot ellipticity of the experimental repeats (n=3, mean ± SD) at 288 nm at the measured pH conditions to determine transitional pH (pH_T_) from the inflection point of the Boltzmann sigmoidal curve. Source data for this figure are provided as a Source Data file.


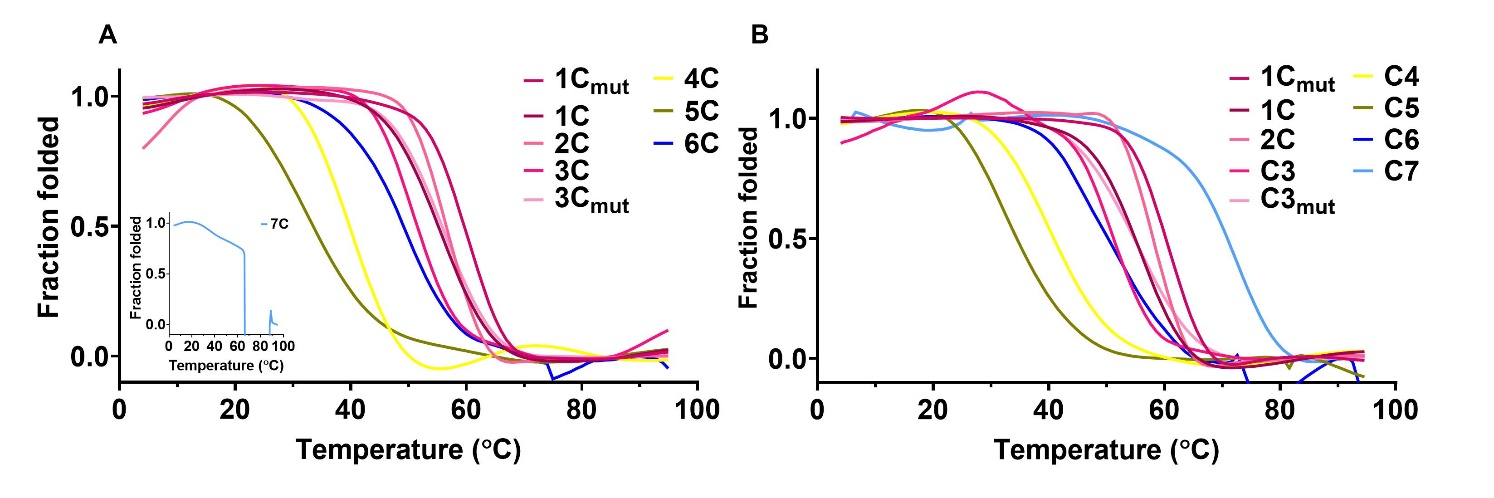


**Figure S3**. Fraction folded UV-melt recorded at 295 nm (A) and 260 nm (B) spectra analysis was performed using 2.5 µM DNA in 10 mM NaCaco 100 mM KCl at pH 5.5. The insert in A shows the lack of melting profile at 295 nm for 7C variants. Source data is available.

**
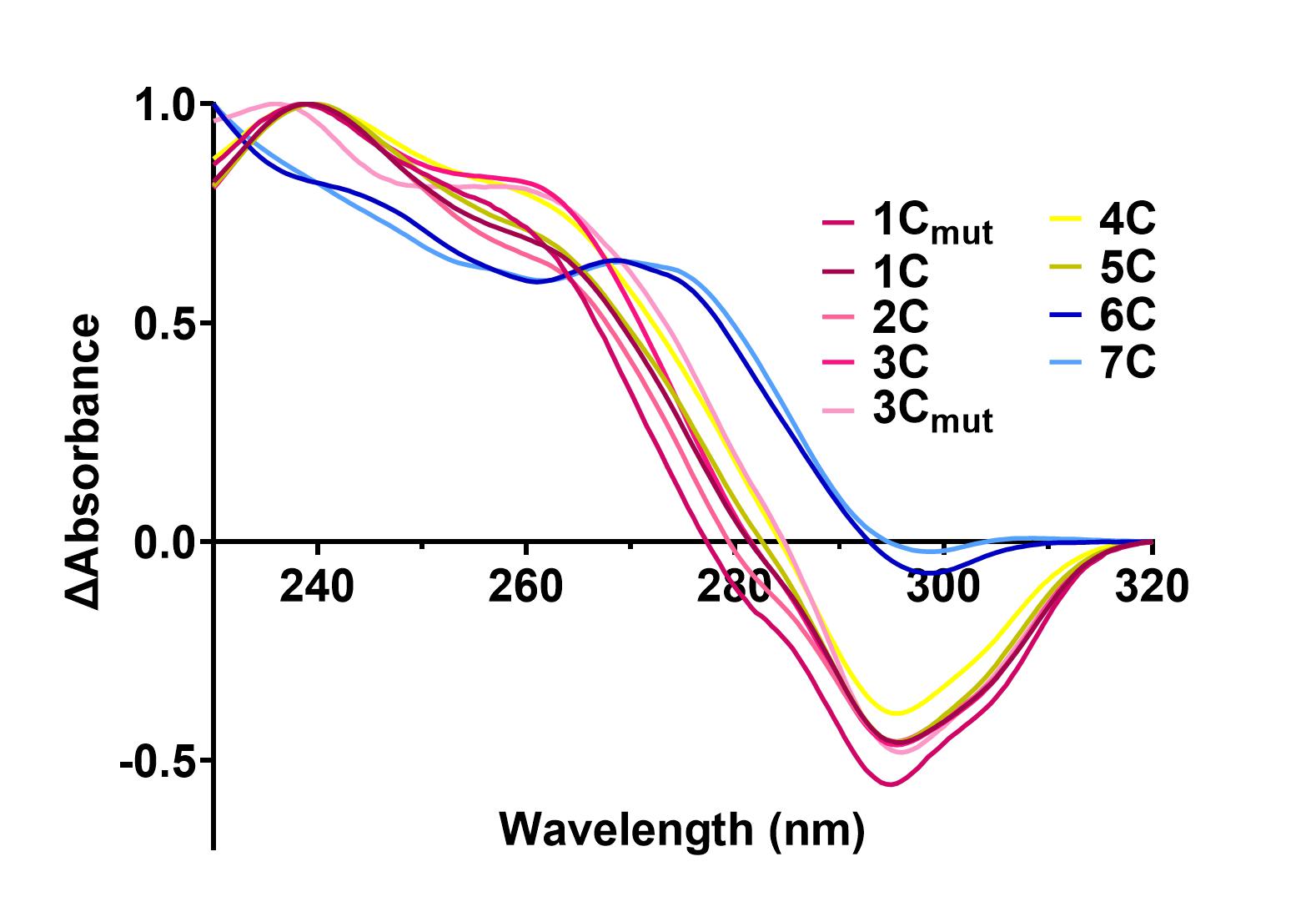
**

**Figure S4.** Thermal Difference Spectra of C-rich ILPR variants published in Guneri *et al*.^1^ for comparison to mutant ILPR C-rich sequences annealed as 2.5 µM DNA in 10 mM NaCaco 100 mM KCl at pH 5.5. Source data is available.

**
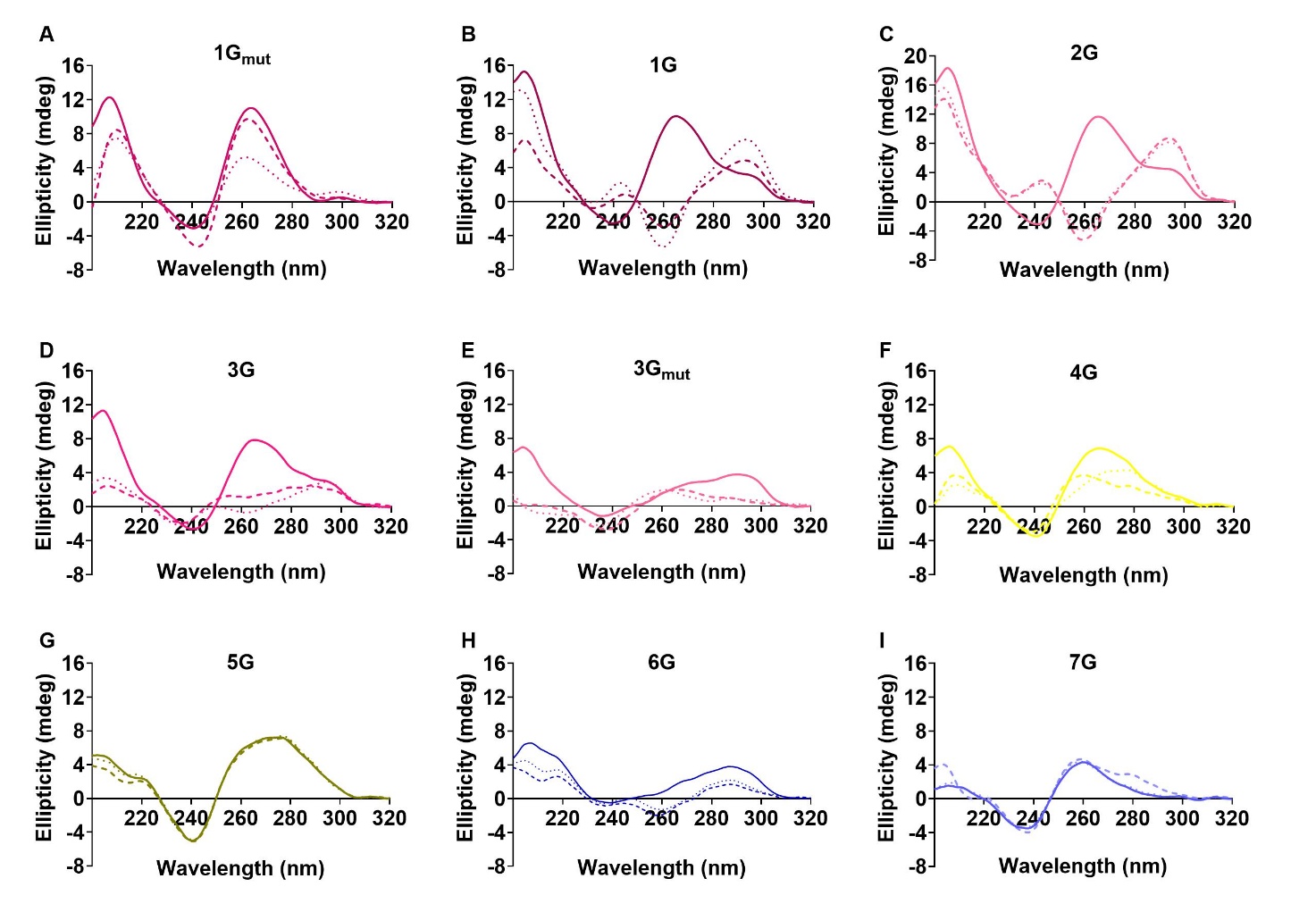
**

**Figure S5.** Biophysical characterisation of G-rich ILPR mutant sequences for non-canonical DNA structure formation and cation dependency. CD spectra analysis was performed using 10 µM DNA in 10 mM NaCaco 100 mM KCl at pH 7.0 (solid line), 100 mM NaCl (dashed line), or 100 mM LiCl (dotted line). Source data is available.


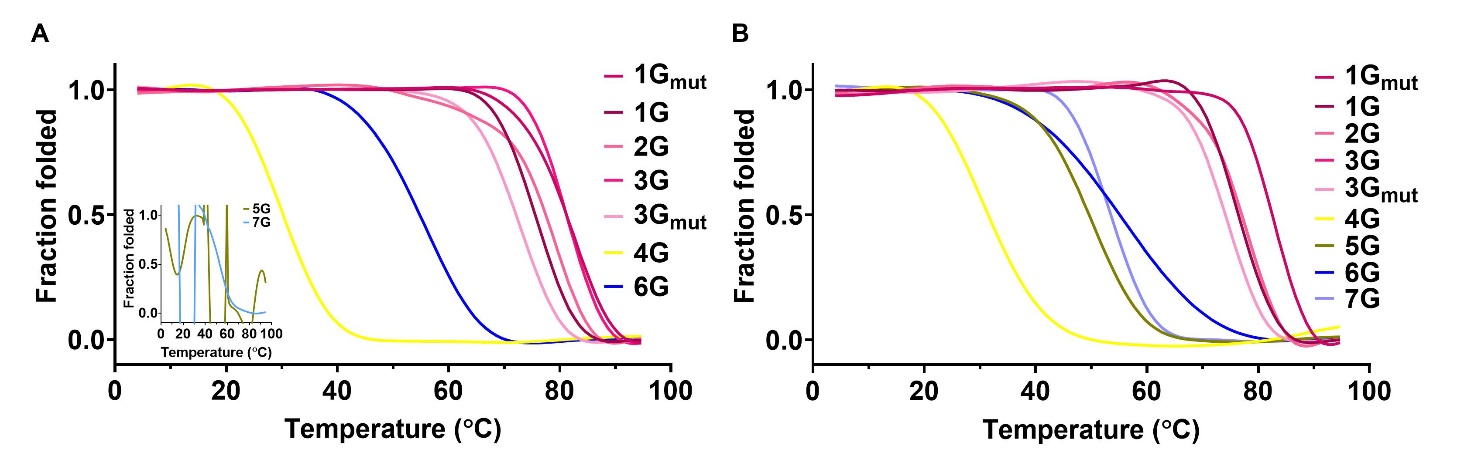


**Figure S6**. Fraction folded UV-melt recorded at 295 nm (A) and 260 nm (B) spectra analysis was performed using 2.5 µM DNA in 10 mM NaCaco 100 mM KCl at pH 5.5. The insert in A shows the lack of melting profile at 295 nm for 5G and 7G variants. Source data is available.


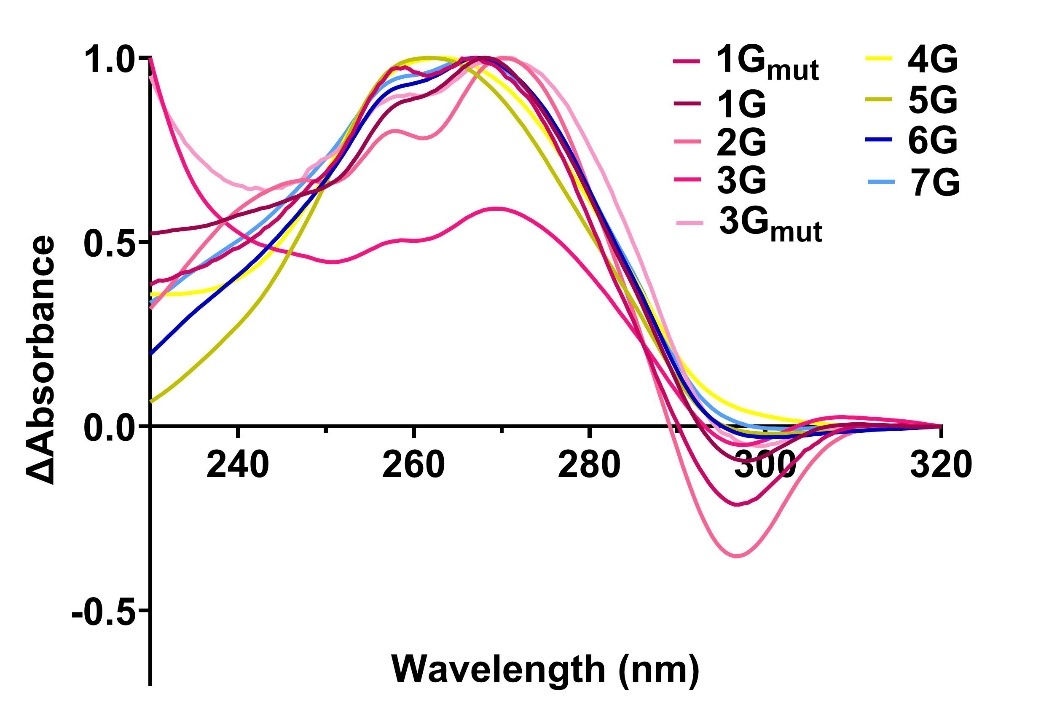


**Figure S7.** Thermal Difference Spectra of G-rich ILPR variants published in Guneri *et al*.^1^ for comparison to mutant ILPR G-rich sequences annealed as 2.5 µM DNA in 10 mM NaCaco 100 mM KCl at pH 7.0. Source data is available.


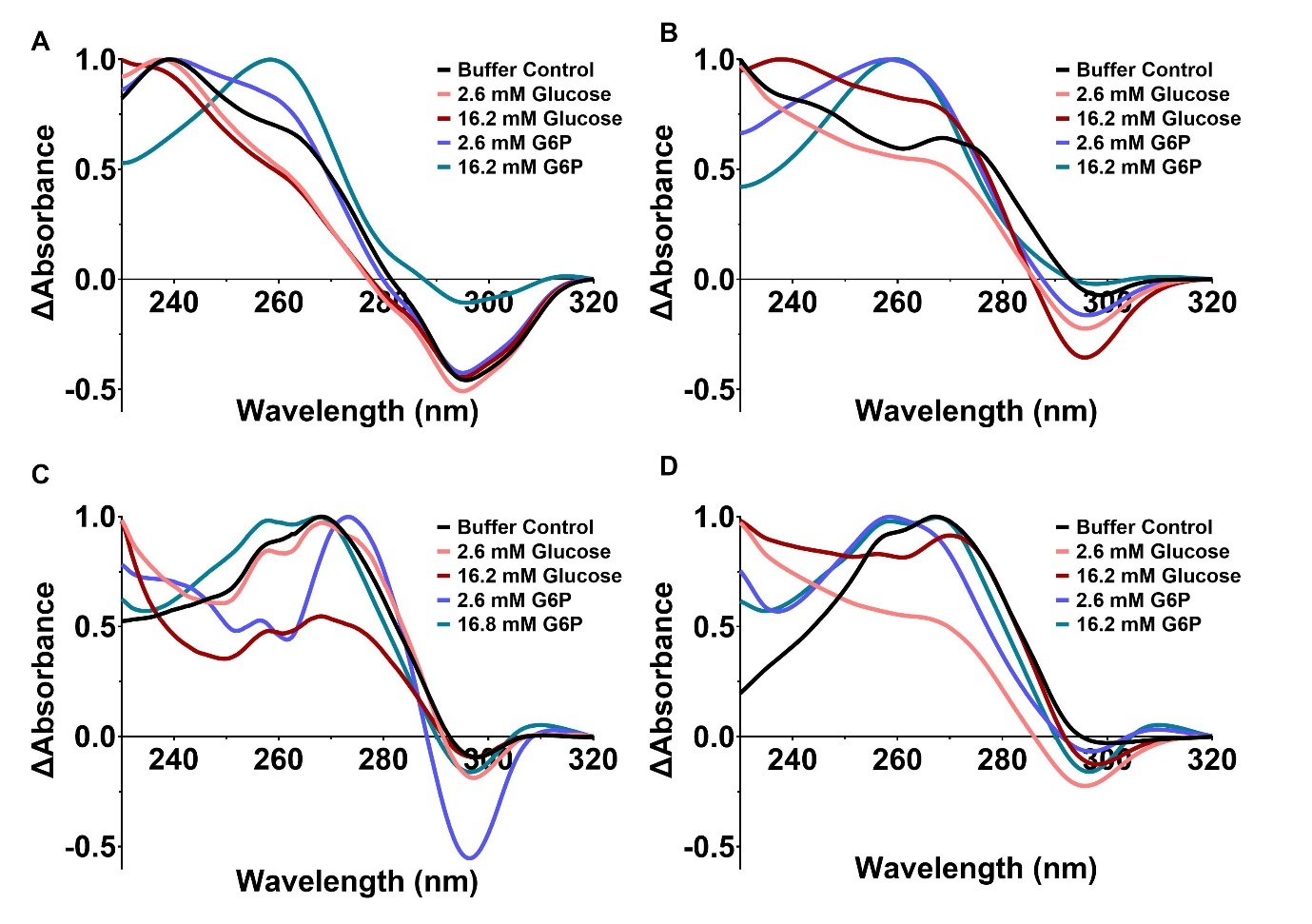


**Figure S8.** Thermal Difference Spectra of ILPR 1C (A) and ILPR 6C (B) and ILPR G1 (C) and ILPR 6G (D) annealed as 2.5 µM DNA in 10 mM NaCaco 100 mM KCl at pH 5.5 for C-rich sequences and pH 7.0 in G-rich sequences and treated with buffer control, 2.8 mM or 16.2 mM glucose or glucose-6-phosphate (G6P). Source data is available.


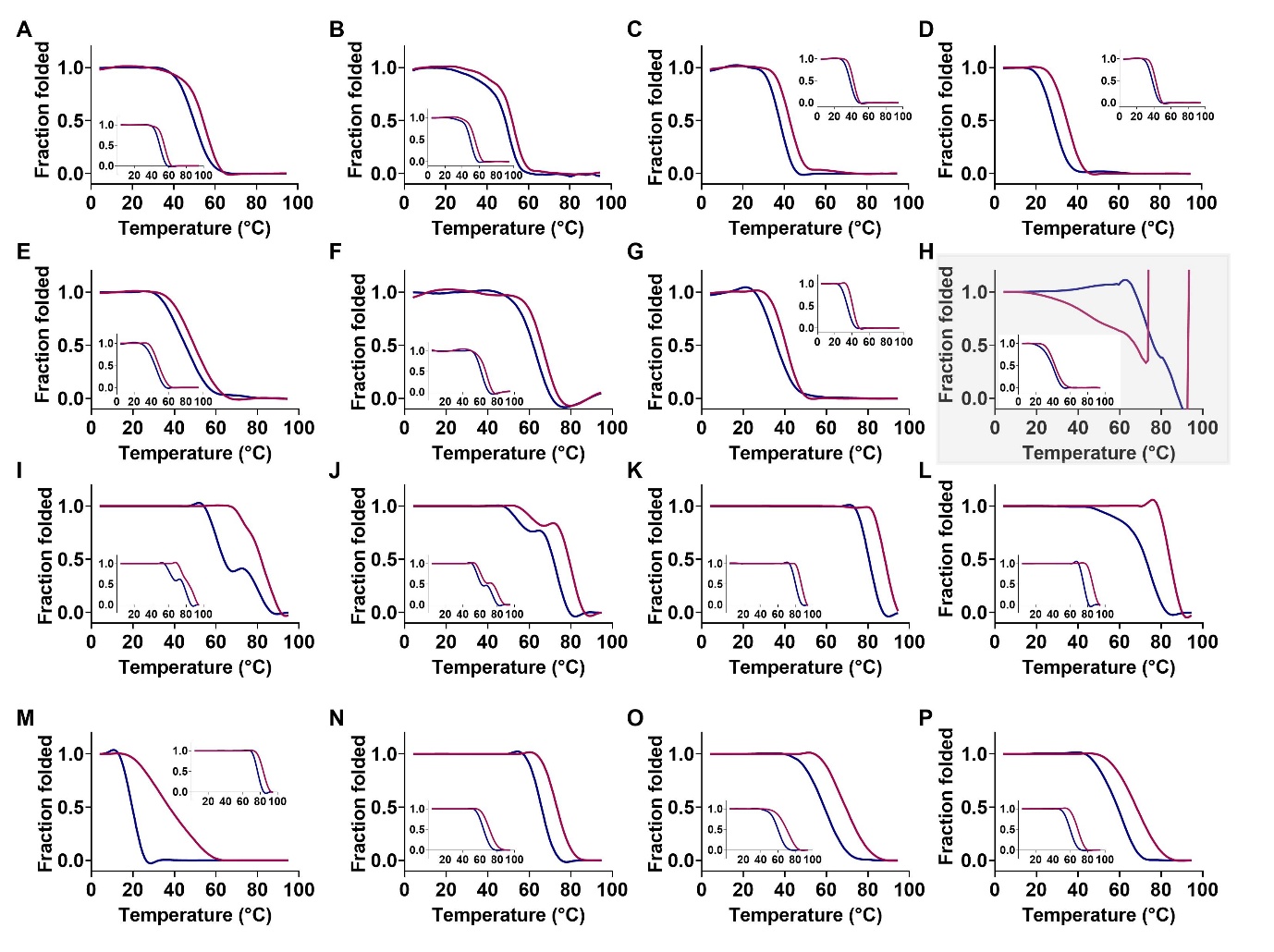


**Figure S9.** Fraction folded UV melting (purple) and annealing (blue) of ILPR C1 (A-2.8 mM glucose, B-16.2 mM glucose, C-2.8 mM glucose-6-phosphate, D-16.2 mM glucose-6-phosphate), ILPR C6 (E-2.8 mM glucose, F-16.2 mM glucose, G-2.8 mM glucose-6-phosphate, H-16.2 mM glucose-6-phosphate), ILPR G1 (I-2.8 mM glucose, J-16.2 mM glucose, K-2.8 mM glucose-6-phosphate, L-16.2 mM glucose-6-phosphate), and ILPR G6 (M-2.8 mM glucose, N-16.2 mM glucose, O-2.8 mM glucose-6-phosphate, P-16.2 mM glucose-6-phosphate) in representative spectra at 295 nm and 260 nm (inserts) of 2.5 µM DNA in 10 mM NaCaco 100 mM KCl at pH 5.5 for C-rich sequences and buffer at pH 7.0 for G-rich sequences. Graphs with grey background showed no repeatable thermodynamic profile. Source data for this figure are provided as a Source Data file.


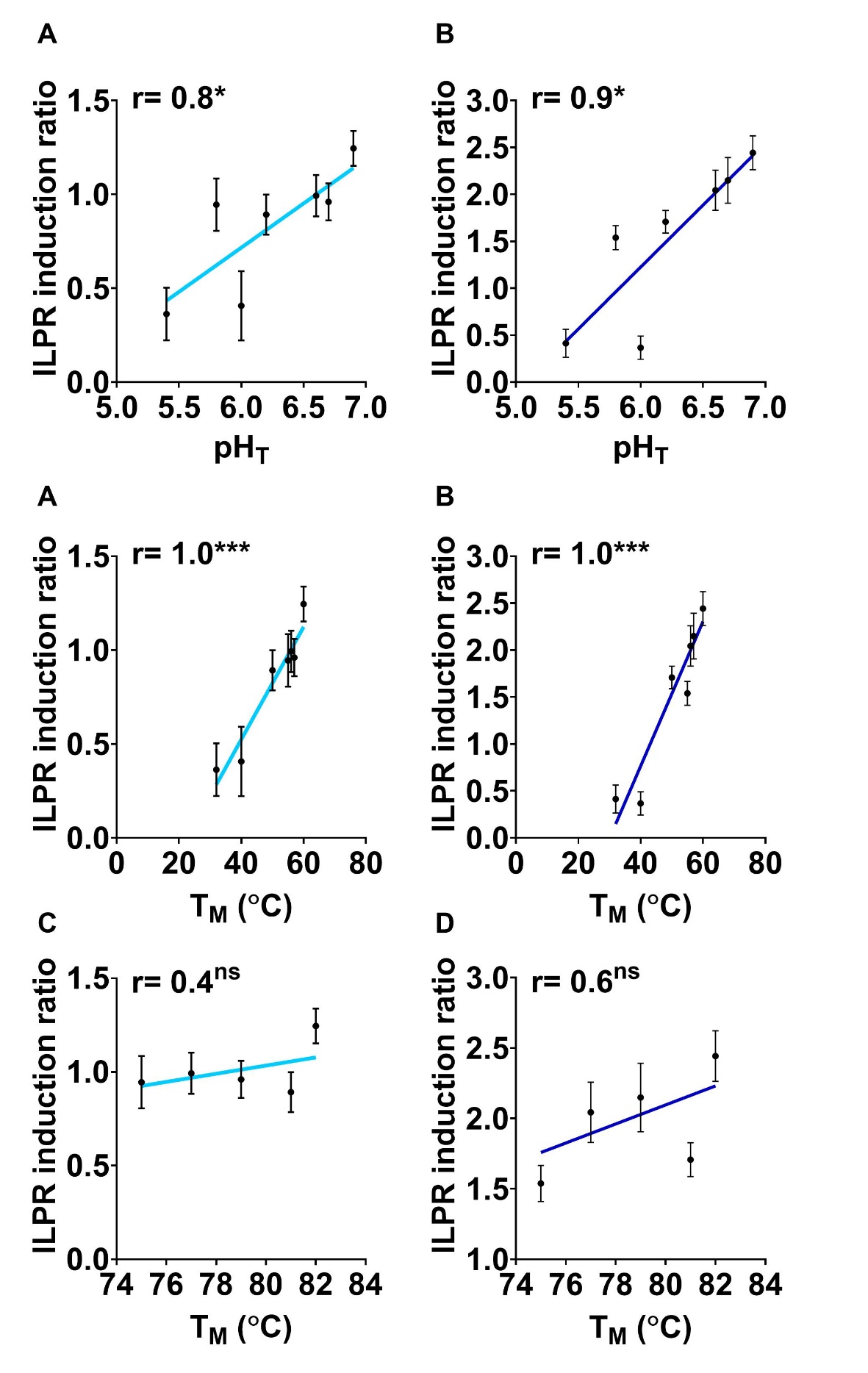


**Figure S10.** Pearson’s correlation between transitional pH of C-rich ILPR variants capable of forming i-motifs and corresponding ILPR induction ratio in dual luciferase reporter gene assay in presence of 2.8 mM Glucose (A) and 16.2 mM Glucose (B). Data shown as Mean ± SD (n=6 for reporter gene assay, n=3 for biophysical data), student t-test , ns > 0.1, p>0.033*, p> 0.002**, p< 0.001***.

**References**

(1) Guneri, D.; Alexandrou, E.; El Omari, K.; Dvořáková, Z.; Chikhale, R. V.; Pike, D. T. S.; Waudby, C. A.; Morris, C. J.; Haider, S.; Parkinson, G. N.; Waller, Z. A. E. Structural insights into i-motif DNA structures in sequences from the insulin-linked polymorphic region. *Nature Communications* **2024**, *15* (1), 7119. DOI: 10.1038/s41467-024-50553-0.
